# Contextual Factors Affecting Risky Decision Making: The Influence of Music on Task Performance and Perceived Distraction

**DOI:** 10.3389/fpsyg.2022.818689

**Published:** 2022-03-02

**Authors:** Melissa T. Buelow, Melissa K. Jungers, Cora Parks, Bonnie Rinato

**Affiliations:** Department of Psychology, The Ohio State University, Newark, OH, United States

**Keywords:** risky decision making, risk perception, music, contextual factors, executive functions

## Abstract

Previous research has investigated factors that contribute to the development of different risk-taking behaviors, such as can occur on lab-based behavioral risky decision making tasks. On several of the most common tasks, participants must develop an adequate understanding of the relative risks and benefits associated with each decision in order to learn to decide advantageously. However, contextual factors can affect the decision making process and one’s ability to weigh the risks and benefits of a decision. The present study investigates the extent to which music may be an additional contextual factor that can disrupt decision making and other executive functions. Across four studies we examine whether having music playing passively in the background or having participants actively listen to music affects performance on measures of risky decision making, working memory, processing speed, and problem solving. Participants reported greater distraction for rock music than classical music in the passive listening studies but did not report any differences in distraction across conditions in the active listening studies. Despite this self-reported increased level of distraction, few significant differences were found in task performance across groups and across studies. The Angling Risk Task (ART) was sensitive to differences in risk by condition, with music leading to greater risk-taking in a passive listening study, but less risk-taking in an active listening study, compared to no music. The extent to which music serves as a contextual factor disrupting performance on measures of risky decision making and other executive functions may depend in part on whether individuals are actively versus passively listening to the music.

## Introduction

Real-world risk-taking and decision making behaviors are increasingly examined *via* performance on lab-based behavioral decision making tasks versus self-reported behavior in hypothetical situations. As more and more behavioral tasks are developed, an understanding of the factors that can affect performance on these tasks is needed. Without an examination of contextual and other factors that can affect performance, researchers (and for some tasks, clinicians) would be unable to determine whether lower scores on tasks are due to true risky decision making and/or risk-taking behaviors or instead to an aspect of the testing situation or participant’s personality/effort/mood/etc. In the present series of studies, we examine the extent to which one contextual factor, music as a distraction during testing, could negatively affect performance across a variety of standard behavioral decision making and executive function tasks.

### Risky Decision Making

Risky decision making can be defined in various ways, and these differences in operational definitions reflect subtle differences in the type of decision making each task assesses. In general, decision making involves a choice between two or more options, but this can be influenced by factors such as the amount of knowledge one has on which to base the decision (Brand et al., 2005; Reyna and Brainerd, 2011), the perceived potential gains or losses associated with each available option (Tversky and Kahneman, 1974; Madden and Bickel, 2010; Reyna and Huettel, 2014), and even one’s emotional state during the decision (Reyna, 2004; Wood and Bechara, 2014). When decisions are made in the face of known risks, or when there are both potential gains and potential losses associated with the available options, risky decision making can occur. In the real world, these decisions often involve a potential negative consequence for one’s health or well-being. Performance on lab-based behavioral tasks, such as the Iowa Gambling Task (IGT; Bechara et al., 1994), Balloon Analog Risk Task (BART; Lejuez et al., 2002), and Game of Dice Task (GDT; Brand et al., 2005), often predicts greater involvement in real-world health-risk behaviors such as lack of seatbelt or helmet use, excessive substance use, and risky sexual behaviors (see Buelow, 2020, for review). To make the safest decision, individuals frequently need to first accurately perceive, then carefully weigh the relative risks and benefits of each available option.

Although performance on these behavioral tasks is associated with real-world risk-taking behavior, other factors, such as personality and demographic characteristics, situational or contextual factors, cognition (and executive functions in particular), and aspects of the tasks themselves can affect decisions and their predictive utility for real-world behaviors. For example, previous research has shown varying effects of gender (Lejuez et al., 2002; Byrne and Worthy, 2016), age (Bishara et al., 2009; Figner et al., 2009; Koscielniak et al., 2016), and the personality characteristics of impulsivity and sensation seeking (Lejuez et al., 2002; Buelow and Suhr, 2013; Buelow, 2015), behavioral activation/inhibition systems (Franken and Muris, 2005; Suhr and Tsanadis, 2007), and narcissism (Brunell and Buelow, 2017) that depend, in part, on the particular behavioral decision making task utilized in each study. Decision making is an executive function (e.g., Clark et al., 2001; Torralva et al., 2009), a group of higher-order cognitive abilities associated with the frontal lobe of the brain (Lezak et al., 2004), but does not consistently correlate with performance on measures of problem solving, abstract reasoning, working memory, or other executive functions (e.g., Romer et al., 2011; Schiebener et al., 2011; Campbell et al., 2013). Providing more detail at the start of the task (e.g., Brand et al., 2005), adding trials (Buelow et al., 2013; Lin et al., 2013), and even changing aspects of the testing situation (e.g., participant mood, effort, and stress; Must et al., 2006; Preston et al., 2007; Okdie et al., 2016) can affect task performance, in some cases by interfering with one’s ability to learn the relative risks and benefits of each decision. We previously demonstrated that taxing working memory by having individuals complete a dual-task paradigm can shift decision making strategies on the IGT (Buelow et al., 2019). Can listening to music, or to someone talking, be a sufficient enough distraction to negatively affect decision making? Do the specifics of the task matter?

### Music and Cognition

The music literature frequently focuses on whether the tempo of the music, the mood the music induces, or the specific type of music affects cognition and real-world risk-taking behaviors. Music can affect cognition (Kellaris et al., 1993), such as by serving as an enhancer or a distractor during a cognitive task (e.g., Wallace, 1994; Thompson et al., 2005; Alley and Greene, 2008; Escoffier et al., 2010; Ferreri et al., 2013; Kang and Williamson, 2013). In a study comparing expert musicians to novices on a visuospatial task presented individually (single task) or at the same time as a music recognition task (dual task), Cocchini et al. (2017) found that the presence of music led to lowered task performance regardless of prior history with music. This finding runs contrary to other studies that instead find an enhancing effect of music training on cognition and on executive functions in particular (Bialystok and DePape, 2009; Ramchandra et al., 2012; Hou et al., 2014; Moradzadeh et al., 2015; Suarez et al., 2016). Others find a history of music training can diminish the negative consequences of the dual-task paradigm (e.g., Moradzadeh et al., 2015).

Some previous research investigated how music affects decision making in lab-based settings. Listening to faster music, compared to slower music, leads to quicker bets (Dixon et al., 2007; Spenwyn et al., 2010; Bramley et al., 2014; Mentzoni et al., 2014) and greater delay discounting (Kim and Zauberman, 2017). However, faster music also leads to more accurate decisions, when the decisions themselves are difficult (versus easy) to make (Day et al., 2009). When different music genres are compared, quicker bets are seen with popular compared to classical music (Bramley et al., 2014) and riskier choices are made during happy versus sad music or random tones (Schulreich et al., 2014). Using music to evoke a happy versus sad mood also affects decisions on the IGT (Shukla et al., 2019); however, when using movie clips instead of music, a fast manipulation led to riskier BART decisions and greater likelihood of risk-taking behavior on a self-report measure (Chandler and Pronin, 2012). Others instead find that the presence of any music, versus none, increases self-reported risk-taking behavior (Enstrom and Schmaltz, 2017). Collectively, music can influence performance on some decision making tasks in lab-based settings, but only a few tasks have been examined to date despite differences between each task.

Music can also affect real-life areas such as casino-based and online gambling. Music can affect gambling behaviors (Griffiths and Parke, 2003, 2005). Background music, particularly loud, slow music combined with casino noises, helps gamblers better estimate time spent gambling compared to casino noises alone (Noseworthy and Finlay, 2009). Music tempo also influences betting speed, with faster music leading to faster betting (Dixon et al., 2007; Spenwyn et al., 2010; Bramley et al., 2014; Mentzoni et al., 2014). However, faster tempo music does not change betting amount relative to slower music (Bramley et al., 2014) and slower music may increase gambling persistence (Mentzoni et al., 2014). The relationship between music, music rate, and gambling behaviors may vary due to aspects of the casino environment (Marmurek et al., 2007) or lighting (Spenwyn et al., 2010), or even based on one’s own perception of whether music affects these behaviors (Bramley et al., 2018). In addition, these effects could be due to mood induction from the music; however, in a study that controlled style and tempo/rate of music, neither rate nor arousal level influenced gambling behaviors (Bramley et al., 2016).

### The Present Study

Although various contextual factors are known to affect performance on some behavioral decision making tasks, it is not known whether music serves as a distraction that negatively affects decision making across different tasks or negatively affects performance on measures of related executive functions. In many of the previous studies of contextual factors affecting task performance, only one behavioral decision making task, commonly the IGT, was included (e.g., Must et al., 2006; Preston et al., 2007; Buelow et al., 2013; Lin et al., 2013; Okdie et al., 2016). However, previous research also suggests that the IGT measures a related but non-overlapping component of the decision making process as other tasks such as the BART, GDT, and Columbia Card Task (CCT; Figner et al., 2009) (e.g., Lejuez et al., 2003a; Aklin et al., 2005; Brand et al., 2007; Buelow and Blaine, 2015). For example, the IGT is thought to measure decision making under ambiguity in the early trials and decision making under risk in the later trials, the BART more explicit risk-taking behavior, the GDT decision making under explicit risk, and the CCT both hot/affective and cold/deliberative aspects of decision making. To more fully assess whether contextual factors affect decision making, multiple assessments of the construct should be included in the same study.

In the present set of studies, we examine the extent to which music (with and without lyrics) and spoken words (podcasts) can serve as a distraction during lab-based behavioral risky decision making and other executive function tasks. Based on previous research indicating that music can lead to increased gambling in casino settings (Marmurek et al., 2007; Noseworthy and Finlay, 2009; Bramley et al., 2014) and that dual-task paradigms can change decision making strategies (Buelow et al., 2019), we hypothesize that individuals in music listening conditions would show riskier (less advantageous) decision making on tasks compared to participants in the control (no music) conditions. We also explore whether the type of music matters, hypothesizing that listening to rock music would lead to riskier decisions than listening to classical music. We present the results of two sets of studies. Studies 1 and 2 focus on how listening to music may serve as a distraction on a series of standard risky decision making tasks. We manipulated the extent to which participants paid attention to the music across these studies, as in Study 1 the music was played passively in the background whereas in Study 2 participants were asked to actively listen to the music. Studies 3 and 4 expand our research to include not just risky decision making tasks, but assessments of other executive functions including problem solving, set shifting, and working memory. As in the first set of studies, participants listened to the music more passively in Study 3 and more actively in Study 4.

## Study 1 Method

### Participants

Participants were 90 undergraduate students at a regional campus of a large Midwestern University. Due to missing data on one of the decision making tasks, eight participants were removed from further analyses, leaving a final sample of 82 participants (*M*_*age*_ = 18.93, *SD*_*age*_ = 2.53, and *Range*_*age*_ = 18–35; 39 males; 68.9% white and 10.8% Black or African American). All were enrolled in psychology courses in which course credit was provided for participation in research studies.

### Measures

#### Balloon Analog Risk Task

The standard BART was administered (Lejuez et al., 2002). The task was created to assess real world risk-taking behavior by having participants pump up a series of 30 balloons, earning 5 cents per pump. But balloons will pop if you pump them up too much and the earned money will be lost. Participants must decide to stop pumping up a balloon before it pops in order to gain the money earned on that balloon. Participants are not told about the probability a balloon will pop, but one of the initial balloons will pop within the first few trials to show participants that this “threat” is real (Lejuez et al., 2002). Thus, participants must weigh the perceived risk associated with losing the gained money on a balloon with the desire to maximize overall profits. Validity for the task is shown *via* correlations with impulsivity and sensation seeking (Lejuez et al., 2002; Hunt et al., 2005) but not intelligence or depression (Lejuez et al., 2002, 2003b), and moderate to high correlations are seen across time points (White et al., 2008; Xu et al., 2013; Buelow and Barnhart, 2018). In the present study, two performance metrics were utilized. First, the average number of pumps per balloon, adjusted for only the unexploded balloons, was calculated (as it is unclear how far participants were willing to go on the exploded balloons). Second, the total number of explosions across the 30 trials was calculated. For each, higher scores indicated a greater level of risk-taking on the task.

#### Iowa Gambling Task

The standard IGT was utilized (Bechara et al., 1994; Bechara, 2007). The IGT was created to assess decision making among patients with frontal lobe damage experiencing real-world decision making impairments yet average performance on standard executive function measures. On this task, participants select 100 cards from one of four decks. Unknown at the start of the task and learned through trial-and-error feedback, each deck has a different level of risk associated with it. With each selection, participants have the chance to win either $50 or $100, but there is a risk of loss on each choice as well. On average, participants earn $100 per selection from Decks A and B and $50 per selection from Decks C and D. After 10 selections from Decks A and B, participants have incurred a net loss of $250. Decks A and B are termed disadvantageous decks, as selections from these decks result in a high immediate reward but even higher losses, resulting in long-term negative outcomes. Decks C and D earn on average $50 per selection and after 10 selections instead incur a net gain of $250. Decks C and D are termed advantageous decks. Selections from these decks result in a low immediate reward but low losses, resulting instead in long-term positive outcomes. However, the decks also differ in their frequency of losses, as Decks A and C experience a loss on 50% of selections and Decks B and D experience losses on just 10% of selections. As the task progresses, participants learn the relative risks and benefits associated with each deck, in turn adjusting their decision making strategy as these risks are perceived. Validity for the task is demonstrated by impaired performance in neurological and psychiatric populations (e.g., Buelow and Suhr, 2009; Buelow, 2020), with concerns about potential practice effects limiting evaluation of test-retest reliability (Tuvblad et al., 2013; Xiao et al., 2013; Buelow and Barnhart, 2018). As previously mentioned, participants do not know much about the relative risks and benefits of each deck at the start of the task, learning this information through feedback after each selection. The early trials, Trials 1–40, constitute decision making under ambiguity. As the task progresses, Trials 41–100, participants are instead able to make their decisions based on knowledge of the relative risks and benefits of each deck. This decision making during later trials is instead decision making under risk (Brand et al., 2007). In the present study, we calculated the number of advantageous (C+D) minus disadvantageous (A+B) selections for each of the five, 20-card blocks of trials. Positive values indicate more advantageous decisions and negative values less advantageous decisions.

### Procedure

All study procedures were approved by the University’s Institutional Review Board and participants first provided informed consent before being randomly assigned to one of two groups: classical music (*n* = 28) or rock music (*n* = 28). Data for participants in the no music condition (*n* = 26) were taken from a separate study taking place at the same time in the lab (no experimental manipulation occurred). All participants completed the IGT and BART in a counterbalanced order. Participants in the two music conditions were told that there would be music playing in the background, but that they did not need to pay attention to the music (i.e., passive music listening). Participants also completed a demographic questionnaire, which included self-report of previous history of taking formal music lessons (excluding voice or chorus). At the end of the study, participants in the music conditions completed a questionnaire assessing if they had heard the musical selections before (0 = *no*, 1 = *yes*), their prior knowledge of the musical selections (0 = *knew none of the songs* to 3 = *knew all of the songs*), and how distracted they were by the music during testing (0 = *not at all* to 3 = *severely*). All participants were then debriefed and course credit assigned.

### Data Analysis

A mixed ANOVA was conducted on the IGT, with block (1–5) as the within-subjects variable and music condition (no music, classical, and rock) as the between-subjects variable. A one-way ANOVA was conducted on the BART. Significant omnibus tests were followed-up with Tukey *post-hoc* analyses. Independent-samples *t-*tests were utilized to assess differences between the music conditions on the end of study questionnaire.

## Study 1 Results

All study means and standard deviations are in Table 1. Participant self-reported gender and age did not differ across groups, *p*s > 0.152, nor were they correlated with the decision making tasks. Therefore, we did not covary these in the remaining analyses.

**TABLE 1:** Study 1 and 2 variable means (standard deviations).

	Study 1			Study 2				
	No music	Classical	Rock	No music	Classical	Rock	Instrumental rock	Podcast
*n*	26	28	28	52	47	48	50	25
Age	18.86 (2.39)	18.65 (1.62)	19.26 (3.30)	18.43 (0.80)	18.73 (1.65)	18.84 (1.97)	19.17 (2.61)	19.52 (5.65)
Gender	10 Males	18 Males	11 Males	26 Males	21 Males	12 Males	26 Males	8 Males
Years music training	–	3.11 (3.19)	2.29 (2.48)	1.26 (2.33)	1.76 (2.32)	1.59 (2.86)	1.29 (2.28)	2.08 (3.29)
IGT								
AD1	–3.08 (8.32)*^a^*	–2.43 (5.92)	–2.07 (6.29)	–2.69 (6.66)	–3.34 (4.49)	–3.67 (4.62)	−3.92 (6.01)	–1.68 (4.92)
AD2	0.85 (7.51)	1.57 (6.19)	–0.14 (7.54)	0.62 (7.86)	0.34 (5.78)	–0.38 (4.49)	–0.16 (5.75)	–0.16 (3.46)
AD3	3.31 (9.00)*^a^*	2.43 (8.39)	0.07 (9.12)	2.81 (7.85)*^a^*	2.13 (7.96)	1.71 (7.25)	–0.54 (7.75)	–2.04 (5.15)
AD4	4.15 (8.20)*^a^*	2.00 (9.19)	1.07 (9.61)	1.04 (9.91)	1.06 (8.10)	–0.04 (6.73)	1.20 (9.14)	–0.12 (8.44)
AD5	4.54 (10.56)*^a^*	2.64 (8.18)	2.89 (11.98)	2.58 (9.89)	0.72 (8.27)	0.38 (5.95)	2.20 (9.28)	0.40 (8.89)
BART								
AAP	33.42(11.44)*^a^*	27.31 (11.34)	29.55 (11.76)	30.69 (11.07)	31.15 (14.64)	30.29 (11.93)	28.30 (12.95)	29.06 (14.08)
#Exp	8.69 (3.93)*^a^*	7.04 (3.82)	7.46 (4.42)	7.81 (3.27)	8.00 (4.37)	7.83 (4.20)	6.71 (3.51)	7.15 (4.53)
CCT	–	–	–	10.69 (1.83)	11.79 (2.96)	11.07 (3.23)	10.84 (4.47)	11.85 (2.89)
GDT	–	–	–	2.37 (10.00)	3.50 (7.57)	0.13 (9.78)	1.17 (8.81)	2.05 (12.07)
Knew	–	0.68 (0.55)	0.25 (0.44)	–	0.79 (0.42)	0.27 (0.45)	0.33 (0.47)	–
Familiar	–	2.10 (0.72)	1.75 (0.71)	–	2.19 (0.48)	1.91 (0.30)	2.41 (0.87)	–
Distract	–	1.14 (0.36)	2.21 (0.96)	–	2.33 (0.87)	2.14 (0.80)	2.34 (0.98)	–

*IGT, Iowa gambling task, advantageous minus disadvantageous (AD) selections by 20-card blocks of trials; BART, Balloon analog risk task, average adjusted pumps (AAP) per balloon and number of explosions (#Exp); CCT, Columbia card task; GDT, game of dice task.*

*^a^TOST indicated potentially meaningful small effects (non-significant p-values) at ±0.44 boundary.*

On the IGT, Mauchly’s test was significant, so the Greenhouse-Geisser correction was applied. There was a significant main effect of Block, *F*(3.46, 273.64) = 8.38, *p* < 0.001, η_p_^2^ = 0.096. Regardless of music condition, participants improved as the task progressed. Block 1 scores were significantly lower than the remaining blocks (*ps* < 0.003), and Block 2 was significantly lower than Block 5 (*p* = 0.020). There was not a significant main effect of Music Condition, *F*(2,79) = 0.53, *p* = 0.591, η_p_^2^ = 0.013, or a Block × Music Condition interaction, *F*(6.93, 273.64) = 0.56, *p* = 0.786, η_p_^2^ = 0.014. There were also no significant differences on the BART for either average adjusted pumps, *F*(2,79) = 1.93, *p* = 0.152, η_p_^2^ = 0.047, or total explosions, *F*(2,79) = 1.19, *p* = 0.309, η_p_^2^ = 0.029.

Examining the end of study questionnaire, significant differences emerged between the music conditions. A greater number of participants in the classical condition reported familiarity with the songs than participants in the rock condition, *t*(54) = 3.22, *p* = 0.002, Cohen’s *d* = 0.50. Participants in the rock music condition reported greater distraction than participants in the classical music condition, *t*(54) = −5.55, *p* < 0.001, *d* = 0.73.

### Exploratory Analyses

Previous research shows a “prominent deck B” phenomenon (e.g., Lin et al., 2007; Chiu et al., 2012; Barnhart and Buelow, 2021) that could lead to lowered scores on the IGT when the standard scoring approach is utilized. Seeking to minimize the frequency of losses is considered an alternate decision making strategy on the task (showcasing a focus on minimizing perceived risks of an immediate loss rather than maximizing long-term totals), meaning that Decks B and D would be considered advantageous and Decks A and C disadvantageous. Utilizing this alternative scoring approach, again no significant Music Condition, *F*(2,79) = 0.64, *p* = 0.531, η_p_^2^ = 0.016, or Block × Music Condition, *F*(5.99, 236.44) = 1.31, *p* = 0.253, η_p_^2^ = 0.032, effects emerged.

To assess whether small sample size might have affected our ability to detect between group differences, we collapsed across the two music conditions to create a no music (*n* = 26) and a combined music (*n* = 56) group. No differences were seen from the previous results, as other than the IGT main effect of Block (*p* < 0.001, η_p_^2^ = 0.099), no between-group differences were found, *p*s > 0.070. Finally, no significant correlations emerged between participants’ years of music lessons and any of the decision tasks, *p*s > 0.156.

Finally, we utilized the two-one-sided tests of significance (TOST) procedure to assess the extent to which these non-significant findings may be due to the effects themselves being very small, versus the absence of an effect (Lakens et al., 2018). We examined the previous literature to find comparisons that most closely matched those in the present study and for which sufficient information was provided to calculate Cohen’s *d*. Mentzoni et al. (2014)’s comparison of betting behaviors as a function of fast and slow tempo music resulted in an effect size of 0.44. Spenwyn et al. (2010)’s comparison of betting speeds as a function of fast and slow tempo music resulted in an effect size of 1.11. We conducted the TOST analyses on our most highly-powered comparisons, those for the combined music and no music conditions. For the TOST analyses, non-significant *p*-values indicate that the observed effect is outside the boundary of −0.44 to 0.44 or −1.11 to 1.11 and may be potentially meaningful. Results of the TOST analyses indicated that nearly all of the comparisons were potentially meaningful at the 0.44 boundary but none were at the 1.11 boundary (Table 1). That leaves us with the possibility that the effect of passive (background) music on decision making may be very small but meaningful.

## Study 1 Discussion

Overall, our findings show limited evidence that music served as a significant distraction during lab-based behavioral decision making tasks. Decision making on the IGT and BART did not vary significantly due to the presence of music, even when the two music conditions were collapsed into one. However, the TOST analyses provided some evidence that the magnitude of the effect may be very small but potentially meaningful. Interestingly, history of music lessons was also not related to task performance despite previous literature indicating a potential benefit on cognitive tasks (e.g., Bialystok and DePape, 2009; Moradzadeh et al., 2015; Suarez et al., 2016). There are several possible explanations for our lack of findings. First, this was a relatively small pilot study, with fewer than 30 participants per group after removing those with missing data. As such, we were only sufficiently powered to detect large effects (*f* > 0.40) with 90% power and α = 0.05, and our demonstrated effect sizes were in the small to moderate range. Second, the music was playing in the background during the testing session. Although it was loud enough to be heard throughout the testing room, it was unclear if participants were actively listening to the music instead of attempting to ignore it while focusing on the decision tasks. It is possible that the music manipulation was not sufficient to induce a dual-task paradigm, which can negatively affect performance on decision making tasks (Pabst et al., 2013a,b; Buelow et al., 2019) and lead to variations in task performance among musicians (e.g., Moradzadeh et al., 2015; Cocchini et al., 2017). Finally, one of our music conditions (classical) was instrumental while the other music condition (rock) combined instrumentation with lyrics. It is possible that the presence/absence of spoken words could affect decision making task performance.

In Study 2, we aimed to take several of these limitations into account. We obtained a larger sample of participants while also expanding our groups to include an instrumental rock music condition and a podcast condition, allowing us to further tease apart the influence of music versus spoken words as a contextual factor. In addition, we created a more active listening component to the study to encourage participants to pay attention to the study manipulation. Finally, we expanded our assessment of risky decision making to include two tasks that focus more on explicit risk, the CCT (Figner et al., 2009) and the GDT (Brand et al., 2005). We hypothesized that participants in the music conditions would show riskier (worse) decision making across tasks than participants in the no music condition. We did not have a specific hypothesis regarding differences between the music groups, instead aiming to examine potential differences in decision making across instrumental and lyric/spoken word selections.

## Study 2 Method

### Participants

Participants were 245 undergraduate students at a regional campus of a large Midwestern University. Due to missing data on either the IGT or BART, 23 participants were removed from further analyses, leaving a final sample of 222 participants (*M*_*age*_ = 18.87, *SD*_*age*_ = 2.59, and *Range*_*age*_ = 18–44; 93 males; 70.6% white and 15.0% Black or African American). All were enrolled in psychology courses in which course credit was provided for participation in research studies.

### Measures

#### Balloon Analog Risk Task and Iowa Gambling Task

The standard BART and IGT were again administered, with total scores calculated in the same manner as in Study 1.

#### Columbia Card Task

On the CCT, participants are tasked with turning over a series of 32 cards (Figner et al., 2009). At the top of the screen is information about the amount won per each gain card (10 or 30 points), the number of loss cards (1 or 3), and the amount to be lost if a loss card is turned over (250 or 750 points). On the “cold” version of this task, participants also see a set of numbers, 0 through 32, at the top of the screen and click on the number to reflect the number of cards to turn over on the trial. They do not receive any immediate feedback about their decision, but are told the total number of points earned at the end of the task. Performance on the CCT is predicted by some executive function tasks (Buelow, 2015) and scores remain moderately correlated across time (Buelow and Barnhart, 2018). In the present study, the average number of cards selected, adjusted for only those trials without a loss card chosen, was calculated. Higher scores indicated greater risky decision making.

#### Game of Dice Task

On the GDT, participants are tasked with predicting the roll of a virtual die (Brand et al., 2007). They are asked to choose a single number ($1000 bet) or a combination of two ($500 bet), three ($250), or four ($100 bet) numbers, before the die is rolled. If the number matches one of the predictions, the participant wins the money. Choosing a one or two number option is considered riskier whereas choose a three or four number option is considered safer. Strong correlations are seen between the GDT and final IGT trials (Brand et al., 2007) and performance is moderately to strongly correlated across time (Buelow and Barnhart, 2018). In the present study, a total net score was calculated by subtracting the number of 1 and 2 number combination selections from the number of 3 and 4 number combination selections. Positive scores indicate more advantageous decisions and negative scores indicate riskier decisions.

### Procedure

All study procedures were approved by the University’s Institutional Review Board and participants provided informed consent. Participants were then randomly assigned to one of five groups: no music (*n* = 52), classical music (*n* = 47), rock music (*n* = 48), instrumental rock music (*n* = 50), or podcast (*n* = 25). All participants completed the decision making tasks in a random order. To create a more active listening environment, participants completed a secondary task during the decision making tasks. Participants in the classical and instrumental rock conditions were asked to pay attention to the selections and count the number of tempo or mood changes. Without writing anything down during the song itself, they were asked to record the number of changes counted at the end of each song selection. Participants in the rock music condition were given a particular word and asked to keep track, again without writing anything down during the song, of the number of times they heard that word in each song. Participants in the podcast condition were told they would be summarizing the podcast at the end of the study. At the end of the study, participants in the music conditions completed the same questionnaire as in Study 1 to assess prior knowledge of and familiarity with the musical selections. Participants in the podcast condition were asked to provide a short, written summary of the podcast. All participants, except for those in the no music condition, were asked how distracted they were by the music condition during testing. Finally, all participants were debriefed and course credit was assigned.

### Data Analysis

A mixed ANOVA was conducted on the IGT, with block (1–5) as the within-subjects variable and music condition as the between-subjects variable. One-way ANOVAs were conducted on the BART, CCT, and GDT. Significant omnibus tests were followed-up with Tukey *post-hoc* analyses. Independent-samples *t-*tests were utilized to assess differences between the music conditions on the end of study questionnaire. Of note, the podcast condition was included as a pilot examination assessing the influence of spoken words compared to music, resulting in a smaller number of participants in this compared to the other conditions. Using G*Power (Faul et al., 2007), we conducted a power analysis to determine the largest effect we were adequately powered to detect. With a power of 0.90 and alpha set to 0.05, we were sufficiently powered to detect a small to moderate effect on the IGT (*f* > 0.12) and moderate effects on the remaining tasks (*f* > 0.26).

## Study 2 Results

All study means and standard deviations are in Table 1. Age was negatively correlated with IGT Block 1 and the GDT. Analyses were conducted with and without age as a covariate, with no significant difference in the pattern of findings. For brevity, we present the results of the findings without age as a covariate. On the IGT, Mauchley’s test was significant, so the Greenhouse-Geisser correction was applied. There was a significant main effect of Block, *F*(3.24, 702.60) = 15.62, *p* < 0.001, η_p_^2^ = 0.067, as participants—regardless of music condition—improved from their Block 1 decisions, *p*s < 0.001. The remaining blocks (2–5) were not significantly different from one another, *p*s > 0.064. There was not a significant main effect of Music Condition, *F*(4,217) = 0.73, *p* = 0.569, η_p_^2^ = 0.013, nor a Block × Music Condition interaction, *F*(12.95, 702.60) = 1.09, *p* = 0.366, η_p_^2^ = 0.020. There were also no significant differences on the BART for either average adjusted pumps, *F*(4,216) = 0.39, *p* = 0.819, η_p_^2^ = 0.007, or total explosions, *F*(4,174) = 0.88, *p* = 0.474, η_p_^2^ = 0.016.

Examining the end of study questionnaire, significant differences emerged in terms of familiarity with the songs and self-reported level of distraction. A greater number of participants in the classical condition reported previous hearing/familiarity with the songs than participants in the rock (*p* < 0.001) or instrumental rock (*p* < 0.001) conditions, *F*(2,132) = 17.05, *p* < 0.001, η_p_^2^ = 0.205. No significant differences emerged between the classical, rock, and instrumental rock conditions in terms of self-reported level of distraction, *F*(2,133) = 0.75, *p* = 0.473, η_p_^2^ = 0.011.

### Exploratory Analyses

Due to computer error, a smaller set of participants completed the CCT (*n* = 143) and GDT (*n* = 147) rendering these analyses exploratory in nature. No significant between-group differences were found on either the CCT, *F*(4,138) = 0.80, *p* = 0.528, η_p_^2^ = 0.023, or the GDT, *F*(4,142) = 0.33, *p* = 0.861, η_p_^2^ = 0.009. Similar to Study 1, we examined the frequency-based scores on the IGT, with no noted differences in the pattern of findings (i.e., no Music Condition, *p* = 0.592, or interaction, *p* = 0.220, effects).

We collapsed across the three music conditions (classical, rock, and rock instrumental) to assess differences in decision making as a function of no music (*n* = 52), music (*n* = 145), and spoken word/podcast (*n* = 25). No differences were seen from the previous pattern of results, as again no between-group differences emerged on the BART, *p*s > 0.781, IGT, *p*s > 0.263 (Block main effect remained significant), CCT, *p* = 0.542, or GDT, *p* = 0.869. Finally, participants’ years of music lessons were positively correlated with decision making on the IGT in Block 3, *r*(219) = 0.133, *p* = 0.049, however no other correlations were significant, *p*s > 0.159.

Finally, we again conducted the TOST analyses to assess the extent to which these non-significant findings may be due to the effects themselves being very small, versus the absence of an effect (Lakens et al., 2018). We utilized the same 0.44 and 1.11 comparison points. In contrast to the first study, all but one TOST analysis was significant, meaning that nearly none of the music/no music comparisons were potentially meaningful at the 0.44 or 1.11 boundaries (Table 1). It is likely that these small effects, in contrast to Study 1, were not meaningful.

## Study 2 Discussion

Collectively, passively (Study 1) or actively (Study 2) listening to music while completing a secondary risky decision making task did not significantly alter performance when the standard scoring approaches were utilized. Contrary to the first study, participants in Study 2 did not report a greater level of distraction as a function of the specific music condition they were randomized to. We found some evidence that a history of music training was associated with performance on the IGT, but this was limited to just one of the five blocks of trials. Our lack of findings across different decision tasks, both those that assess explicit risk perception and those that assess risk perception learned through trial-and-error feedback, provide some evidence that music, whether presented in the background during a testing session or as part of a dual-task paradigm, is not sufficient to alter decision making on these lab-based tasks.

However, the present study is also not without limitation. Although we increased our sample size compared to Study 1, the number of participants in the podcast condition was lower by study design. We also had fewer participants complete the CCT and GDT compared to the IGT and BART, which could have affected our ability to detect group differences on these tasks. Finally, both of these studies were very specifically focused on how music as a contextual factor may affect performance on behavioral decision making tasks that differ in how risks are presented to participants. Decision making is an executive function and is linked with ventromedial prefrontal cortex functioning. However, other executive functions instead tap into functioning of dorsolateral prefrontal cortex and other areas of the frontal lobe (Lezak et al., 2004). In addition, behavioral task performance does not always correlate with performance on measures of other executive functions (e.g., Romer et al., 2011; Schiebener et al., 2011; Campbell et al., 2013). Accurate and/or less risky decision making does, however, rely on other cognitive skills such as attention, working memory, processing speed, and other executive functions such as problem solving (e.g., Weber and Johnson, 2009; Toplak et al., 2010; Reyna and Brainerd, 2011). It is possible that music may serve as a distraction on other executive function tasks, rather than on those assessing risk perception and risky decisions. If this is the case, we should see lower performance on executive function tasks but not risky decision making tasks in the music condition compared to the control condition. We added one additional risky decision making task, the Angling Risk Task (ART; Pleskac, 2008), to Studies 3 and 4 to test this prediction. The ART was designed to assess both a similar risk perception construct as the IGT and BART and a different aspect of decision making: learning from sequential feedback in the context of explicit information provided at the start of the task. Music could distract the participant from understanding and interpreting this explicit information, lowering performance on the task.

In Study 3, we aimed to replicate and expand our findings from Studies 1 and 2. We expanded our assessment to examine whether music as a contextual factor affects not just risky decision making, but measures of processing speed, working memory, and problem solving. We hypothesized that participants in the music conditions will show worse performance on the study tasks than participants in the no music condition, while again having no specific hypothesis regarding differences between music and lyric/spoken word conditions.

## Study 3 Method

### Participants

Participants were 289 undergraduate students at a regional campus of a large Midwestern University. Due to missing data on one of the study tasks, 31 participants were removed from further analyses. This left a final sample of 258 participants (*M*_*age*_ = 18.66, *SD*_*age*_ = 1.07, and *Range*_*age*_ = 18–25; 109 males; 68.6% white and 14.0% Black or African American). All were enrolled in psychology courses in which course credit was provided for participation in research studies.

### Measures

#### Angling Risk Task

The Angling Risk Task (ART; Pleskac, 2008) was created as a correlate to the BART but with a greater emphasis on how learning affects decision making. Participants take part in a 30-round fishing tournament where they receive five cents per red fish caught but lose all the earned money if a blue fish is caught. Just like on the BART, participants must decide to stop fishing to bank the earned money on a given trial. We utilized the “cloudy day” version of the task to mimic the BART as closely as possible. On this version, the number of red and blue fish is not visible at the start of the task. We calculated the total number of sudden ends (i.e., a blue fish was caught) and the average fish count, adjusted for only those rounds without a sudden end. Riskier decision making is seen with a higher number of adjusted catches and a greater number of sudden ends.

#### Digit Symbol Coding

Digit Symbol Coding (DSC) measures psychomotor processing speed (Wechsler, 2008). Participants view a series of symbols that are matched to different numbers, as well as a series of boxes where the top part has a number but the symbol is missing. They are then tasked with drawing the corresponding symbol below each number as quickly and accurately as possible. The total number of correct responses and total number of errors were calculated, with greater correct responses and fewer errors indicating better psychomotor processing speed.

#### N-Back Task

The N-Back task (e.g., Kirchner, 1958) assesses working memory capacity. On the version utilized in the present study (based on Jaeggi et al., 2010), participants viewed a series of different geometric designs, one at a time. They were tasked with pressing a key every time the current shape matched the shape presented 2-back. For the present study, we calculated the total number of hits (correct responses) and false alarms, with a greater number of hits and fewer false alarms indicating better working memory task performance.

#### Self-Ordered Pointing Task

On the Self-Ordered Pointing Task (SOPT; Petrides and Milner, 1982), a second measure of working memory, participants are tasked with selecting a series of 6, 8, 10, and 12 images without selecting one they previously chose. Complicating this process is the fact that the order of the images rotates with each image selection. For the present study, we calculated the total number of errors collapsed across the four type of trials (6, 8, 10, and 12 image sets), with fewer errors indicating greater working memory task performance.

#### Wisconsin Card Sort Task

The Wisconsin Card Sort Task (WCST; Heaton et al., 1993) assesses problem solving, abstract reasoning, and perseveration. Participants see a set of four key cards and are tasked with matching a larger set of cards to the key cards. They are not told how to match the cards, instead learning from trial-and-error feedback after each choice. For the present study, we calculated the total number of categories completed (maximum 6), total number of perseverative errors (e.g., errors in which the participant continued to match cards to an outdated sorting principle), and the total number of failures to maintain set (e.g., participant made an error after correctly sorting cards at least five times in a row). Better performance on the task is associated with more categories completed, fewer perseverative errors, and fewer failures to maintain set.

### Procedure

All study procedures were approved by the University’s Institutional Review Board and participants first provided informed consent. They were then randomly assigned to one of three groups: no music (*n* = 115), classical music (*n* = 70), or rock music (*n* = 73). Of note, the no music participants were oversampled due to the nature of the larger study. All participants completed the tasks in a randomized order. Just as in Study 1, participants in the two music conditions heard music playing in the background during testing (i.e., passive music listening). At the end of the study, the music condition participants completed a questionnaire assessing if they had heard the musical selections before (0 = *no*, 1 = *yes*), prior knowledge of the musical selections (0 = *knew none of the songs* to 3 = *knew all of the songs*), and self-reported level of distraction during testing (0 = *not at all* to 3 = *severely*). All participants were then debriefed and course credit was assigned.

### Data Analysis

A series of one-way ANOVAs were conducted on the ART, DSC, NB, SOPT, and WCST outcome variables. Significant omnibus tests were followed-up with Tukey *post-hoc* analyses. Independent-samples *t*-tests were utilized to assess differences between the music conditions on the end of study questionnaire. We again assessed the largest effect we were adequately powered to detect. With a power of 0.90 and alpha set to 0.05, we were sufficiently powered to detect small to moderate effects across tasks (*f* > 0.22).

## Study 3 Results

All study means and standard deviations are in Table 2. Gender was correlated with scores on the ART and N-Back; however, as this was not our primary variable of interest and no differences were found in the pattern of results with or without this covariate, we present the results without the covariate. There were significant between-group differences in the average adjusted number of catches on the ART, *F*(2,255) = 3.43, *p* = 0.034, η_p_^2^ = 0.026. Participants in the no music condition had fewer catches than participants in the rock (*p* = 0.031) or classical (*p* = 0.029) conditions, with no difference between the two music conditions (*p* = 0.966). A similar pattern emerged for the number of sudden ends, *F*(2,255) = 8.53, *p* < 0.001, η_p_^2^ = 0.063. Participants in the no music condition had fewer sudden ends than those in the rock (*p* < 0.001) or classical (*p* = 0.025) conditions, with no difference between the two music conditions (*p* = 0.476). On the WCST, there were no group differences in total number of categories completed, *F*(2,255) = 0.23, *p* = 0.794, η_p_^2^ = 0.002, or total perseverative errors, *F*(2,255) = 1.64, *p* = 0.195, η_p_^2^ = 0.013. However, participants in the no music condition had fewer failures to maintain set than those in the rock music condition (*p* < 0.001), *F*(2,255) = 6.77, *p* = 0.001, η_p_^2^ = 0.050. No group differences emerged in failures to maintain set across no music and classical music (*p* = 0.430) or rock and classical music (*p* = 0.080) conditions. The remaining comparisons indicated no significant between-group differences on DSC [total correct responses: *F*(2,255) = 1.64, *p* = 0.197, η_p_^2^ = 0.013; total errors: *F*(2,255) = 0.63, *p* = 0.534, η_p_^2^ = 0.005], N-Back [total hits: *F*(2,255) = 0.51, *p* = 0.604, η_p_^2^ = 0.004; false alarms: *F*(2,255) = 0.10, *p* = 0.909, η_p_^2^ = 0.001], or SOPT [total errors: *F*(2,255) = 1.12, *p* = 0.329, η_p_^2^ = 0.009].

**TABLE 2:** Study 3 and 4 variable means (standard deviations).

	Study 3			Study 4			
	No music	Classical	Rock	No music	Classical	Rock	Podcast
*N*	115	70	73	114	77	77	73
Age	18.83 (1.06)	18.60 (1.25)	18.52 (0.85)	18.63 (1.20)	18.76 (1.59)	18.85 (1.47)	18.59 (1.08)
Gender	53 Males	31 Males	25 Males	60 Males	34 Males	36 Males	24 Males
Years Music Training	1.54 (3.12)	2.09 (2.96)	2.08 (3.12)	1.79 (2.95)	2.16 (3.35)	1.81 (2.73)	1.63 (2.69)
ART							
Ends	6.64 (4.05)	8.50 (5.36)	9.41 (4.90)	10.46 (5.01)	9.78 (4.64)	10.17 (5.05)	10.10 (4.93)
Avg	25.23 (14.73)	30.27 (16.31)	30.16 (14.86)	35.90 (17.80)	29.84 (14.99)	30.26 (15.54)	35.52 (17.39)
DSC							
Correct	61.22 (16.23)	63.40 (17.74)	58.37 (16.41)	62.14 (18.93)	53.71 (18.55)	55.49 (20.45)	61.38 (18.67)
Errors	3.76 (13.09)	2.27 (3.14)	2.74 (4.46)	1.96 (2.50)	1.97 (2.13)	2.05 (4.81)	1.82 (2.04)
N-Back							
Hits	26.83 (10.49)	27.67 (10.32)	28.36 (9.83)	26.25 (10.54)	25.53 (10.02)	23.29 (8.42)	25.33 (11.30)
FA	33.58 (20.67)	34.51 (23.41)	34.92 (20.75)	32.45 (21.16)	33.74 (20.20)	34.91 (14.29)	32.25 (21.74)
SOPT							
Errors	13.64 (13.95)	13.11 (13.96)	16.77 (21.04)	15.53 (17.32)	17.14 (20.90)	20.73 (21.63)	20.19 (22.15)
WCST							
Cat	4.96 (1.68)	4.97 (1.69)	5.11 (1.25)	4.96 (1.50)	4.78 (1.68)	4.64 (1.71)	4.71 (1.66)
PE	7.67 (4.55)	7.11 (3.10)	8.36 (4.23)	7.79 (3.00)	8.61 (6.37)	8.45 (3.59)	7.71 (3.67)
FMS	0.81 (1.01)	1.04 (1.33)	1.49 (1.47)	1.28 (1.37)	1.25 (1.15)	1.70 (1.83)	1.32 (1.29)
Knew	–	0.78 (0.42)	0.55 (0.50)	–	0.79 (0.41)	0.33 (0.47)	–
Familiar	–	1.30 (0.76)	1.03 (0.51)	–	0.95 (0.40)	1.11 (0.74)	–
Distract	–	0.58 (0.71)	1.06 (0.85)	–	1.33 (1.02)	1.62 (0.92)	–

*ART, Angling risk task, number of sudden ends (Ends) and average adjusted casts (Avg); DSC, Digit symbol coding, number correct (Correct) and number of errors (Errors); N-Back, N-Back task, number of hits (Hits) and number of false alarms (FA); SOPT, Self-ordered pointing test, number of errors; WCST, Wisconsin card sort task, number of categories completed (Cat), number of perseverative errors (PE), and number of failures to maintain set (FMS).*

*^a^TOST indicated potentially meaningful small effects (non-significant p-values) at ±0.44 boundary.*

Examining the end of study questionnaire, significant differences emerged in terms of familiarity with the songs and self-reported level of distraction. A greater number of participants in the classical condition reported familiarity with the songs than participants in the rock condition, *F*(1,123) = 7.71, *p* = 0.006, η_p_^2^ = 0.059. In addition, participants in the rock condition reported a higher level of distraction than those in the classical condition, *F*(1,123) = 11.67, *p* < 0.001, η_p_^2^ = 0.087.

### Exploratory Analyses

We again conducted additional exploratory analyses with the conditions collapsed into no music (*n* = 115) and any music (*n* = 143) groups. On the ART, the no music condition had a significantly lower average adjusted number of catches than the combined music condition, *t*(256) = −2.63, *p* = 0.009, *d* = 0.33. In addition, those in the no music condition had a fewer number of sudden ends than those in the combined music condition, *t*(256) = −3.96, *p* < 0.001, *d* = 0.50. Collapsing across music condition did not change results for DSC total correct, *t*(256) = 0.86, *p* = 0.855, *d* = 0.02, or total errors, *t*(256) = 1.08, *p* = 0.280, *d* = 0.13; N-Back total hits, *t*(256) = −0.93, *p* = 0.356, *d* = 0.12, or false alarms, *t*(256) = −0.42, *p* = 0.672, *d* = 0.05; SOPT total errors, *t*(256) = −0.65, *p* = 0.513, *d* = 0.08; or WCST total categories, *t*(256) = −0.43, *p* = 0.502, *d* = 0.05, or total perseverative errors, *t*(256) = −0.15, *p* = 0.879, *d* = 0.02. Those in the no music condition experienced fewer failures to maintain set than those in the combined music condition, *t*(256) = −2.96, *p* = 0.003, *d* = 0.37. Finally, participants’ years of music lessons were not correlated with any of the executive function tasks, *p*s > 0.071.

We again conducted the TOST analyses based on the 0.44 and 1.11 boundaries. None of the previously non-significant findings were potentially meaningful at the 0.44 or 1.11 levels (Table 2). Thus, it is not likely that the non-significant findings on the DSC, N-Back, and SOPT reflect very small but meaningful effects.

## Study 3 Discussion

Overall, our findings show evidence that music can affect performance on some—but not all—executive functions assessed. Specifically, both rock and classical music were associated with a greater number of catches and more sudden ends (e.g., riskier decisions) on the ART and more failures to maintain set (e.g., lowered monitoring) on the WCST. We failed to find evidence of music group-based differences on measures of working memory or processing speed, nor that music lessons affected performance on any of the tasks. It is unclear why we failed to find group differences on the BART in Study 1 but found group differences on the ART in Study 3 as they follow a similar format. It is possible that our larger sample sizes here allowed us to detect these relatively small between-group effects.

Just as with Study 2, we conducted Study 4 to determine if using music to create a dual-task paradigm negatively affected performance on an expanded battery of executive function tasks. We hypothesized that participants in the music conditions would show worse executive task performance than those in the no music condition, while not making specific hypotheses about how each music condition would affect task performance. We also added a full, versus pilot, podcast condition to further examine whether the presence of lyrics/spoken words affected task performance.

## Study 4 Method

### Participants

Participants were 384 undergraduate students at a regional campus of a large Midwestern University. Due to missing data on one of the study tasks, 43 participants were removed from further analyses. This left a final sample of 341 participants (*M*_*age*_ = 18.70, *SD*_*age*_ = 1.33; 154 males; 64.3% white and 20.4% Black or African American). All were enrolled in psychology courses in which course credit was provided for participation in research studies.

### Measures

The same tasks from Study 3 were utilized in Study 4, with scoring following the previously outlined approaches.

### Procedure

All study procedures were approved by the University’s Institutional Review Board and participants first provided informed consent. They were then randomly assigned to one of four groups: no music (*n* = 114), classical music (*n* = 77), rock music (*n* = 77), or podcast (*n* = 73). Of note, the no music participants were oversampled due to the nature of the larger study. All participants completed the tasks in a randomized order. The dual-task paradigm followed that of Study 2. While completing the test battery, participants also kept track of the number of tempo or mood changes (classical condition), number of times a lyric was heard in a song (rock condition), or the context of the podcast in order to write a summary. At the end of the study, participants in the music conditions completed the same questionnaire as in the previous studies to assess prior knowledge of and familiarity with the musical selections. Participants in the podcast condition were asked to provide a written summary of the podcast. All participants, except for those in the no music condition, were asked about their level of distraction during testing. Finally, participants were debriefed and course credit was assigned.

### Data Analysis

A series of one-way ANOVAs were conducted on the ART, DSC, NB, SOPT, and WCST outcome variables. Significant omnibus tests were followed-up with Tukey *post-hoc* analyses. Independent-samples *t*-tests were utilized to assess differences between the music conditions on the end of study questionnaire. With a power of 0.90 and alpha set to 0.05, we were sufficiently powered to detect small to moderate effects across tasks (*f* > 0.20).

## Study 4 Results

All study means and standard deviations are in Table 1. Gender was positively correlated with ART adjusted catches and WCST total categories. Analyses were conducted with and without gender as a covariate, with no significant differences in the pattern of findings. For brevity, we present the results of the findings without gender as a covariate. On the ART, those in the classical (*p* = 0.014) and rock (*p* = 0.022) conditions had significantly fewer catches (less risk-taking) than those in the no music condition, *F*(3,337) = 3.33, *p* = 0.020, η_p_^2^ = 0.029. In addition, the classical condition had fewer catches than the podcast condition (*p* = 0.037). There were no between-group differences in sudden ends, *F*(3,337) = 0.30, *p* = 0.823, η_p_^2^ = 0.003. Participants in the no music condition had more correct responses on DSC than participants in the classical music condition (*p* = 0.016), *F*(3,337) = 4.16, *p* = 0.006, η_p_^2^ = 0.036, with no differences between the remaining groups, *p*s > 0.069. In addition, no group differences were found for DSC total errors, *F*(3,337) = 0.07, *p* = 0.974, η_p_^2^ = 0.001. Similarly, no group differences were found for N Back total hits, *F*(3,337) = 1.35, *p* = 0.260, η_p_^2^ = 0.012, or false alarms, *F*(3,337) = 0.32, *p* = 0.809, η_p_^2^ = 0.003. No group differences emerged in the total errors on the SOPT, collapsed across the four trial lengths, *F*(3,337) = 1.36, *p* = 0.254, η_p_^2^ = 0.012. On the WCST, there were no group differences in failures to maintain set, *F*(3,337) = 1.76, *p* = 0.154, η_p_^2^ = 0.015; total perseverative errors, *F*(3,337) = 0.96, *p* = 0.412, η_p_^2^ = 0.008; or total number of categories completed, *F*(3,337) = 0.73, *p* = 0.537, η_p_^2^ = 0.006.

Examining the end of study questionnaire, significant differences emerged in terms of familiarity with the songs and self-reported level of distraction. A greater number of participants in the classical condition reported familiarity with the songs than participants in the rock (*p* < 0.001) condition, *F*(1,104) = 28.43, *p* < 0.001, η_p_^2^ = 0.215. However, there was not a significant group difference in self-reported distraction across the music conditions, *F*(1,138) = 3.05, *p* = 0.083, η_p_^2^ = 0.022.

### Exploratory Analyses

Similar to the previous studies, we conducted additional exploratory analyses with the conditions collapsed into no music (*n* = 114), any music (*n* = 154), and podcast (*n* = 73) groups. On the ART, the combined music group had a significantly lower average adjusted number of catches than the no music (*p* = 0.005) and podcast (*p* = 0.021) conditions, *F*(2,338) = 4.99, *p* = 0.007, η_p_^2^ = 0.029 (podcast = no music, *p* = 0.879). There were no between-group differences in sudden ends, *F*(2,338) = 0.34, *p* = 0.715, η_p_^2^ = 0.002. On the DSC, the combined music group had significantly fewer correct than the no music (*p* = 0.002) and podcast (*p* = 0.013) conditions, *F*(2,338) = 6.09, *p* = 0.003, η_p_^2^ = 0.035 (podcast = no music, *p* = 0.792). No differences emerged in DSC total errors, *F*(2,338) = 0.10, *p* = 0.906, η_p_^2^ = 0.001. Collapsing across music condition did not change results for N Back total hits, *F*(2,338) = 1.07, *p* = 0.343, η_p_^2^ = 0.006, or false alarms, *F*(2,338) = 0.42, *p* = 0.659, η_p_^2^ = 0.002; SOPT total errors, *F*(2,338) = 1.44, *p* = 0.238, η_p_^2^ = 0.008; or WCST total categories, *F*(2,338) = 0.94, *p* = 0.391, η_p_^2^ = 0.006; total perseverative errors, *F*(2,338) = 1.42, *p* = 0.244, η_p_^2^ = 0.008; or failures to maintain set, *F*(2,338) = 0.68, *p* = 0.508, η_p_^2^ = .004. Finally, participants’ years of music lessons were positively correlated with average adjusted pumps on the ART, *r*(304) = 0.147, *p* = 0.010; N-back total hits, *r*(304) = 0.149, *p* = 0.009; WCST total categories, *r*(304) = 0.124, *p* = 0.031; but negatively correlated with errors on the SOPT, *r*(304) = −0.133, *p* = 0.020 (remaining correlations *ps* > 0.112).

We again conducted the TOST analyses based on the 0.44 and 1.11 boundaries. None of the previously non-significant findings were potentially meaningful at the 0.44 or 1.11 levels (Table 2). Thus, it is not likely that the non-significant findings on the N-Back, SOPT, and WCST reflect very small but meaningful effects.

## Study 4 Discussion

Overall, our findings show evidence that music can affect performance on some—but not all—executive functions assessed, and that how the music is presented, passively in the background versus as part of a dual-task paradigm, can influence this relationship. When music was passively playing in the background (Study 3), those in the two music conditions were riskier on the ART and had fewer failures to maintain set on the WCST compared to those in the no music condition. But when the music was part of a dual-task paradigm (Study 4), those in the music conditions were instead *less risky* on the ART, had more errors on DSC, and did not differ on the WCST from those in the no music condition. Having a background distraction versus having the music as part of a focused task altered how music affected risky decision making on the ART, problem solving on the WCST, and processing speed on DSC. Years of music training were correlated with a performance on four of the five study tasks, indicating some support from previous research showing music training can influence performance during dual-task paradigms. Participants with a greater number of years of music training took greater risks on the ART, but also had more correct responses on the N-Back and WCST and fewer errors on the SOPT, indicating a difference between how previous music training affected risky taking behavior versus the other executive functions.

## General Discussion

The present study sought to further our knowledge of how contextual factors affect performance on behavioral decision making tasks that assess different aspects of risk perception. Previous research has shown changing aspects of the testing situation, such as participants’ mood, effort, stress, or knowledge about the task (e.g., Must et al., 2006; Preston et al., 2007; Buelow et al., 2013; Lin et al., 2013; Okdie et al., 2016) can affect performance on behavioral decision making tasks, as can factors such as personality, gender, and age (e.g., Lejuez et al., 2002; Franken and Muris, 2005; Suhr and Tsanadis, 2007; Bishara et al., 2009; Figner et al., 2009; Buelow and Suhr, 2013; Buelow, 2015; Byrne and Worthy, 2016; Koscielniak et al., 2016; Brunell and Buelow, 2017). Across four studies, we tested whether music playing passively in the background (Studies 1 and 3) or used as part of a dual-task paradigm (Studies 2 and 4) was sufficient to negatively affect performance on measures of decision making and other executive functions.

When music played in the background during testing, no significant differences in task performance were found on the BART or IGT (Study 1), but music was associated with riskier decisions on the ART compared to when no music was playing in the background (Study 3). The results of our TOST analyses indicate that even the non-significant findings may reflect very small but meaningful effects of music on decision making. In addition, music negatively affected the ability to stay focused during the WCST, leading to a greater number of failures to maintain set compared to the no music condition. These findings are consistent with previous research that suggests background music can influence gambling behaviors, risk taking, and performance on various cognitive tasks (Griffiths and Parke, 2003, 2005; Bramley et al., 2016; Enstrom and Schmaltz, 2017; Kim and Zauberman, 2017).

However, a different pattern of findings was seen when music served as part of a dual-task paradigm as participants were instructed to actively listen to the musical selections while also completing the test battery. Although this dual-task paradigm did not lead to a different pattern of decision making on the risky decision tasks in Study 2, those participants in the music conditions in Study 4 made *less risky* decisions on the ART and had more correct responses on DSC than those in the no music condition. Although inconsistent with some of our findings when music played passively in the background, these findings are consistent with some previous research indicating music can enhance performance on cognitive tests (e.g., Wallace, 1994; Day et al., 2009; McMahon et al., 2011; Ferreri et al., 2013; Kang and Williamson, 2013; Halko et al., 2015; Bramley et al., 2016).

Interestingly, the type of music used as the distraction may not matter. Collapsing across the rock and classical music conditions did not change the pattern of results across our studies, contrary to previous research suggesting differences in task performance as a function of popular versus classic music or faster versus slower tempo music (e.g., Day et al., 2009; Dixon et al., 2007; Halko and Kaustia, 2012; Bramley et al., 2014; Mentzoni et al., 2014; Kim and Zauberman, 2017). In addition, although participants reported the rock music was more distracting than the classical music in Studies 1 and 3, no differences in task performance was seen between rock and classical conditions in these studies. The podcast condition was not significantly different from the other conditions in Studies 2 and 4, providing some evidence that it is the music, not spoken words/lyrics, that can distract during testing.

Finally, we also assessed the possibility that an individual differences variable, history of music lessons/training, could affect ask performance. No relationships were found between task performance and music training in our two passive listening studies, but when music served as part of a dual-task paradigm (Studies 2 and 4), participants with more years of music training had better performance on measures of decision making and other executive functions. This finding is consistent with previous research suggesting that music training can diminish the negative consequences of a dual-task paradigm (e.g., Moradzadeh et al., 2015).

Overall, our pattern of findings suggests that music can be viewed as a contextual factor influencing performance on some measures of risky decision making and other executive functions, as the results indicate the type of task matters. Specifically, music influenced performance on a measure of risky decision making that required participants to interpret explicitly presented information at the start of the task and utilize feedback from each trial to learn to decide advantageously within this context (ART). However, the extent to which individuals are actively listening to the music, versus passively listening to it in the background, may make the difference in music serving as a distraction during testing. In addition, we found that just because an individual self-reported being distracted by the music during testing did not mean that scores on the tasks significantly decreased due to the music. One’s prior experience with music training could be one factor affecting the relationship between self-reported distraction and performance on cognitive tasks. A second factor could be working memory capacity, as lowered working memory is associated with decreased performance on other cognitive tasks (e.g., Toplak et al., 2010; Buelow et al., 2019). Or, the presence of music could lead to faster thought (e.g., Dixon et al., 2007; Spenwyn et al., 2010; Bramley et al., 2014; Mentzoni et al., 2014), a process that can lead towards a greater number of errors being made (e.g., Busemeyer and Townsend, 1993; Wenzlaff et al., 2011), due to insufficient information gathering (e.g., Heitz, 2014; Rae et al., 2014), or riskier decisions being made on behavioral tasks (e.g., Vadham et al., 2007; Chandler and Pronin, 2012). Finally, decision making and other task outcomes, when music is present as a contextual factor, may depend in part on how much information is provided directly to participants versus learned through trial-and-error feedback. Future research should investigate these factors to allow for a better understanding of how music could affect task performance.

### Limitations

The present study is not without several limitations. Each study was conducted with an undergraduate student population, only a portion of whom self-reported previous music training experience. We also had relatively small sample sizes in several of the studies, leading to some exploratory analyses with the CCT, GDT, and podcast conditions. It is possible we were not sufficiently powered to detect small effects across these studies. The podcast condition was not part of the initial study conceptualization, leading to a lack of this condition in the passive listening studies. We were therefore unable to assess whether having a podcast playing in the background during testing negatively affected task performance. We also cannot be certain that participants who reported being familiar with or knowledgeable about the classical music selections were truly familiar with these selections. Finally, although we utilized more than one behavioral risky decision making or executive function task in each study, it is still possible that the use of other tasks could indicate music served as a greater distraction than we say in this series of studies.

## Conclusion

The effect of music on decision making, executive functions, and perceived risk is complex. The role of music, whether in the background or as a point of focus, changes its effect on subsequent task performance. For example, when participants passively listened, they reported rock music as more distracting relative to classical music, but this did not translate into poorer or more risky performance. Background music in general (both classical and rock) did influence decision making and problem solving, leading to greater risk-taking on the ART and worse performance on the WCST. However, when an active music task was part of the study, participants took fewer risks. These nuanced results based on music condition demonstrate that risky decision making depends, at least in part, on contextual factors. Not only are participants’ decisions influenced by mood, effort, or personality, but also their decisions can be affected by the testing environment and secondary tasks. Clinicians should keep in mind that perceived distraction may not change performance and tasks that assess decision making and executive function are subject to the influence of contextual factors that may go relatively unnoticed in clinical settings.

## Data Availability Statement

The raw data supporting the conclusions of this article will be made available by the authors, without undue reservation.

## Ethics Statement

The studies involving human participants were reviewed and approved by The Ohio State University IRB. The patients/participants provided their written informed consent to participate in this study.

## Author Contributions

MB (Studies 1–4), MJ (Studies 2–4), CP (Study 1), and BR (Study 2) designed the studies. MB analyzed data and drafted the initial manuscript. MJ, CP, and BR collected data and provided revision of the manuscript. All authors contributed to the article and approved the submitted version.

## Conflict of Interest

The authors declare that the research was conducted in the absence of any commercial or financial relationships that could be construed as a potential conflict of interest.

## Publisher’s Note

All claims expressed in this article are solely those of the authors and do not necessarily represent those of their affiliated organizations, or those of the publisher, the editors and the reviewers. Any product that may be evaluated in this article, or claim that may be made by its manufacturer, is not guaranteed or endorsed by the publisher.
